# Assessment of Clinical Hematology Laboratory Performance Using Sigma Metrics and Associated Factors in Total Testing Process at the Dessie Comprehensive Specialized Hospital, Northeast, Ethiopia: A Cross‐Sectional Study

**DOI:** 10.1002/hsr2.72063

**Published:** 2026-03-10

**Authors:** Zewudu Mulatie, Endris Ebrahim, Mihreteab Alebachew, Alemu Gedefie, Bruktawit Eshetu, Mihret Tilahun, Habtu Debash, Yeshimebet Kassa, Ermiyas Alemayehu, Tesfaye Gessese, Dereje Mengesha Berta

**Affiliations:** ^1^ Department of Medical Laboratory Sciences, College of Medicine and Health Sciences Wollo University Dessie Ethiopia; ^2^ Department Hematology and immunohematology, school of biomedical and laboratory science, college of medicine and health science University of Gondar Gondar Ethiopia

**Keywords:** analytical errors, hematology laboratory performance, post‐analytical errors, pre‐analytical errors, sigma metrics, sigma metricssigma metricssigma metrics

## Abstract

**Background and Aims:**

Clinical laboratory test results play a fundamental role in clinical decision making, influencing approximately 70% of medical decisions. Numerous studies have shown that errors can occur throughout the total testing process. This study aimed to assess the performance of the Clinical Hematology Laboratory and identify associated factors using sigma metrics across the total testing process at Dessie Comprehensive Specialized Hospital.

**Methods:**

A cross‐sectional study was conducted from July1, 2023, to September 30, 2023. The study included all eligible laboratory samples and corresponding test requests during the study period using a consecutive sampling technique. Data for each variable were collected using a pre prepared checklist and record formats by trained laboratory professionals. The data were entered into EPI data version 3.1 and exported to Stata version 17 for analysis. Logistic regression analysis was performed, and the laboratory's total testing process performance was evaluated using the sigma scale.

**Result:**

The overall prevalence of clinical hematology laboratory errors in the total testing process was 12.44%. Most errors occurred during the pre‐analytical phase (77.8%), followed by the post‐analytical phase (19.77%) and the analytical phase (2.36%). The overall sigma value of the hematology laboratory was 2.7, indicating substandard performance. The sigma values for the pre‐analytical, analytical, and post‐analytical phases were 2.6, 3.2, and 2.8, respectively.

**Conclusion and Recommendation:**

This study found that errors in the clinical hematology laboratory were common, and overall performance was below the acceptable sigma threshold. Therefore, hospital administration should provide need‐based training for all staff involved in hematology laboratory sample collection and processing. Strengthening adherence to standard operating procedures and implementing continuous quality improvement strategies are essential to enhance laboratory performance and reduce errors.

**Abbreviations**
CBC: complete blood count
CLIA: clinical laboratory improvement amendment
HCT: hematocrit
IPD: Inpatient Department
ISO: International Organization for Standardization
MRN: medical record number
OPD: outpatient department
QIs: quality indicators
TAT: turnaround times
TTP: Total testing process
WHO: World Health Organization

## 1 Introduction

### 1.1 Background

According to research, the utilization of clinical laboratory test results in clinical decision making has become an essential component of medical practice [1, 2]. Approximately 70% of clinical decisions related to hospitalization, admission, prescription, and release depend on laboratory data; however, slight variations between 60% and 80% have been reported [1, 3, 4]. In clinical medicine, screening, diagnosis, and therapeutic monitoring for many diseases and disorders rely heavily on hematology laboratory results. Therefore, ensuring quality in laboratory services is crucial throughout the total testing process (TTP) to produce accurate, precise, and timely results for optimal patient care [5, 6, 7]. The total laboratory testing process is complex and encompasses all steps from the medical decision to perform a test to the delivery of the results. It consists of three main phases: pre‐analytical, analytical, and post‐analytical [8, 9].

Patient safety is a major global public health concern [10]. A Study in Malaysia reported a diagnostic error rate of 3.6% [11]. Similarly, data from the United States indicate that diagnostic errors occur in approximately 5% of outpatient cases [12, 13]. Moreover, evidence from low and middle‐income countries suggests that about 134 million adverse events and 2.6 million deaths occur annually due to medical errors. Among low income countries, South Asia and Western Sub‐Saharan Africa had the highest mortality rates, with 1.9 million and 1.2 million deaths, respectively [14, 15].

Laboratory errors contribute to several clinical problems, including delayed diagnosis, additional laboratory testing, misdiagnosis, overmedication, under‐medication, incorrect treatment, increased healthcare costs, decreased patient satisfaction, injury, and death [16, 17]. Consequently, the quality of laboratory services has become a primary concern for many governments worldwide [18, 19].

In Africa, the magnitude of laboratory errors is higher than in other continents due to inadequate health care infrastructure, a lack of trained personnel, poor management systems, substandard quality control measures, equipment shortages, weak government policies, low staff motivation, and poverty [20, 21]. As a result, most clinical laboratory services in Africa remain below standard, and laboratory participation in accreditation programs is below the expected level [22]. According to ISO15189 reports, the performance of African laboratories is poor. Similarly, the majority of laboratories in Ethiopia are not accredited and fail to meet quality standards. However, laboratory errors still receive little attention and remain difficult to address [22, 23, 24, 25].

Recently, several laboratories worldwide have begun applying sigma metrics principles across all phases of TTP to improve quality, reduce defects, streamline the registration process, decrease patients' wait times, lower costs, and enhance customer satisfaction [26]. During pre‐analytical phases, sigma metrics help improve the quality of information on test requisitions, specimen collection, patient identification, and transportation. In the analytical phase, it aids e in minimizing laboratory testing errors, misinterpretations, misreading and misjudgments of results [27]. Although quality control (QC) programs and quality assurance measures have been established to identify defects, these alone are insufficient for providing a direct and integrated assessment of laboratory process performance [3, 28]. Therefore, sigma metrics serve as an essential tool for evaluating laboratory performance and achieving intended quality goals [28].

Sigma metrics is a quantitative tool used to assess laboratory process performance by measuring defects per‐million opportunities (DPMO) [29]. It is more effective for evaluating laboratory errors than simply counting defects, as sigma metrics analysis assesses the quality of the TTP by providing mechanisms for error correction and performance improvement through models such as the Define, Measure, Analyze, Improve, and Control model. Additionally, sigma metrics help determine the appropriate frequency and number of quality control measures required in a laboratory [30, 31].

Despite efforts to improve performance in African countries, significant challenges persist. Many clinical laboratories continue to provide sub‐optimal services, and the overall quality of their outputs remains poor [25, 32]. Evidence from Ethiopia also supports this concern [25]. However, very few studies have been conducted to assess the overall distribution of clinical hematology laboratory errors, and limited research has evaluated process performance by using sigma metric in Ethiopia, specifically in the study area. We hypothesized that the clinical hematology laboratory's TTP demonstrates suboptimal performance, with sigma metrics below the acceptable threshold, indicating the need for targeted quality improvement interventions. Therefore, this study aimed to assess the performance of the clinical hematology laboratory's TTP using sigma metrics.

## 2 Materials and Methods

### 2.1 Study Area, Design and Period

A cross‐sectional study was conducted at the Clinical Hematology Laboratory of Dessie Comprehensive Specialized Hospital from July 1, 2023, to September 30, 2023. The Hospital laboratory consists of multiple sections, including Phlebotomy, Hematology, Parasitology, Clinical Chemistry, Urinalysis, Serology, Emergency and Microbiology. Among these, the Hematology laboratory serves as the primary unit, performing tests such as complete blood cell count (CBC), Hematocrit (HCT), peripheral morphology, body fluid analysis, erythrocyte sedimentation rate (ESR) and coagulation tests. The Clinical Hematology Laboratory processes approximately 70,000 samples annually.

### 2.2 Study Population

The study included all phlebotomists, laboratory professionals, and laboratory test requests along with their respective blood samples submitted for analysis.

### 2.3 Inclusion and Exclusion Criteria

#### 2.3.1 Inclusion Criteria

Test requests accompanied by blood samples for Clinical Hematology Laboratory analysis were included.

#### 2.3.2 Exclusion Criteria

Tests requested for non‐routine Hematology Laboratory tests were excluded. In addition, tests requiring reagents that were inconsistently available during the study period were also excluded.

### 2.4 Variables

#### 2.4.1 Dependent Variables

Clinical Hematology laboratory performance and frequency of errors.

#### 2.4.2 Independent Variables

Sample collection site, work shift and adherence to SOP.

### 2.5 Operational Definitions

**Clinical laboratory error**: Any defect or mistake occurring at any phase of the total testing process.

**Pre‐analytical errors:** Mistakes that occur before sample analysis.

**Analytical errors:** Errors that arise during testing or analysis.

**Post‐analytical errors:** Mistake that occur after analysis or testing [33].

**Total error:** The sum of all errors that may occur throughout the entire testing process [34].

**Critical values:** Results that fall significantly above or below the reference range and require immediate medical intervention.

**Hemolysis:** The destruction of red blood cells either in vitro or vivo, leading to visibly red plasma in an EDTA‐anticoagulated, settled or centrifuged blood sample.

**Lipemic sample:** Plasma with a visible milky appearance due to high lipid content.

**Clotted sample**: Plasma in a solidified state that may obstruct analyzer probes [35].

**In a sufficient sample:** A collected sample with a volume lower than the acceptable limit.

**Overfilled sample:** A collected sample exceeding the acceptable volume.

**Delayed sample:** A sample left at room temperature for more than 2 h without being analyzed.

**Turnaround time:** The duration between sample collection and the release of test results to the physicians.

**Phlebotomist:** A laboratory or healthcare professional responsible for collecting blood specimens.

**Work shift**: The period during which the Clinical Laboratory is fully operational. It follows a two shift system, each lasting 4 h (1st shift from 8:00 to 12:30 AM and 2nd shift from 1:00 to 5:00 PM).

**Sigma metrics:** A measure of laboratory performance that indicates how many standard deviations (SDs) fit within the tolerance limit from the assay mean. A test is considered unacceptable if the total observed error exceeds the total allowable error. In this study, laboratory performance was evaluated based on the sigma value, as detailed in the table below [36, 37] (Table 1).

**Table 1:** Sigma value interpretations table.

Sigma value	Defect per million opportunities	Yield	Level
1	690,000	30.9	Unacceptable
2	308,537	69.1	Poor
3	66,807	93.3	Marginal
4	6210	99.4	Good
5	233	99.98	Excellent
6	3.4	99.99966	World class

**Unacceptable Test Performance**: The average sigma value is less than or equal to three [37].

**Acceptable Test Performance**: The average sigma value will be greater than three [37].

### 2.6 Sample Size Determination and Sampling Technique

The study included all eligible Clinical Hematology Laboratory samples and their corresponding test requests collected during the study period. A consecutive sampling technique was used to include all test requests submitted for Clinical Hematology Laboratory testing.

### 2.7 Data Collection and Laboratory Methods

Data were collected using a standardized, pre‐tested checklist designed to evaluate errors in the TTP within the Clinical Hematology Laboratory. The checklist was developed based on variables from previous studies and accreditation standards from the College of American Pathologists [17, 35, 38]. After finalizing the checklist, trained data collectors and supervisors were assigned.

All data collectors were laboratory professionals with training in laboratory quality management systems. They were instructed on how to collect relevant data to assess the completeness of standard laboratory request forms, including patient age, gender, physician's signature, location, date and authorized request format. Additionally, they were trained on how to evaluate specimen quality, as well as analytical and post‐analytical quality indicators (QIs) based on the checklist. Data collectors were assigned to sample collection and Hematology laboratory sections, where they systematically recorded observations across all TTP phases. Additionally, face‐to‐face interviews were conducted to collect socio demographic data from phlebotomists and laboratory personnel. Pre‐analytical variables related to laboratory specimen handling were assessed through direct observation upon sample arrival at the Clinical Hematology Laboratory. Post‐analytical variables were recorded after analysis, ensuring that all checklist data were systematically entered.

### 2.8 Quality Assurance

To ensure data quality, a standardized observational checklist was used. A pre‐test was conducted on 40 samples with their corresponding test requests at Borumeda Hospital to assess feasibility and ensure the validity of study tools. Based on the pre‐test findings, necessary modifications were made to the checklist. Furthermore, data quality was ensured by having data collectors verify the completeness of the checklist in real time while gathering relevant information at each phase of the Clinical Hematology laboratory testing process. Supervisors provided daily feedback and corrections to investigators throughout the data collection period. Additionally, the collected data was regularly reviewed for completeness, accuracy, and clarity. Data analysis, reporting, and interpretation adhered to the Guidelines for Reporting Statistics for Clinical Research in Urology [39], SAMPL guidelines [40], and the STROBE checklist for observational studies [41].

### 2.9 Data Analysis and Interpretation

Data was coded and entered to EPI data version 3.1 for completeness and validation before being exported to Stata version 17 for analysis. Descriptive statistics were used to summarize the overall distribution of errors in the Clinical Hematology Laboratory. The association between laboratory performance and the explanatory variable was assessed using bivariable and multivariable logistic regression models. Explanatory variables with a *p*‐value < 0.25 in bivariable analysis were included in the multivariable logistic regression model to control for potential confounders. Model fitness was assessed using the Hosmer–Lemeshow goodness‐of‐fit test. Crude odds ratio (COR) and adjusted odds ratio (AOR) within the 95% confidence interval (CI) were used to determine the strength of associations. The *p*‐value < 0.05 was considered statistically significant.

The laboratory test process was assessed using the Sigma scale, which quantifies errors and defects in the total testing process. This approach monitors each phase, counts errors per million opportunities, and converts them into sigma metrics using a standard sigma conversion table. The calculation steps included:

1. DPMO = (Number of defects × 1,000,000)/number of defect opportunities
2. Convert DPMO to Sigma level: first, find the probability of a defect occurring: P = DPMO/1,000,000
3. Compute *Z*‐score: *Z*= Φ^−1^(1 − P). Find the *Z* value from a *Z*‐table.
4. Convert *Z*‐score to Sigma level: Σ=1 + Z
5. 0.8406

Additionally, sigma values were calculated using the formula: Sigma= (TEa – Bias %)/CV. So, the bias of all parameters was calculated using the following formula: Bias (%) = [(our laboratory mean of IQC data ‐ target mean of IQC data)/target mean of IQC data] ×100. The CV for all parameters was calculated from the observed mean and SD of the daily IQC in the study period by using the following formula: CV (%) = (SD/mean) x 100. In addition, the overall observed error committed within the laboratory was calculated as: TE= Bias (%) + CV (%) × 2. For, improved accuracy, sigma scores were calculated using an online sigma calculator (Defects Per Million Calculator, available at

https://www.westgard.com/six-sigma-calculators.htm).

### 2.10 Ethical Consideration

Ethical approval was obtained from the College of Medicine and Health Sciences Research and Ethics Review Committee of Wollo University. The objective and purpose of the study were explained to the medical director, and a permission letter was obtained from Dessie comprehensive specialized Hospital before the actual data collection began. After providing an explanation of the possible benefits and risks, written informed consent was obtained from study participants. To ensure confidentiality of data, study subjects were identified using codes, and only authorized people had access to the collected data. Identified errors were linked to the responsible people for better patient management and quality improvement.

## 3 Result

### 3.1 General Information

During the study period, a total of 12,402 hematology tests were requested. Complete blood count tests accounted for the majority, comprising 9728 (78.44%) of the total, followed by coagulation tests with 1547 (12.47%). Of all the test requests, 6207 (50.05%) were requested from outpatient department (OPD), while 2550 (20.56%) were from the MCH unit. Most test requests were ordered during the morning shift (57.32%) and, 8255 (66.56%) of samples were collected by laboratory professionals. All sample records were documented manually (Table 2).

**Table 2:** General information for the hematology laboratory at Dessie Comprehensive Specialized Hospital, 2023.

Variables		Frequency	Percentage
Total test request assessed		12,402	100
Test requested for CBC		9728	78.44
Test requested for ESR		680	5.48
Test requested for Manual HCT		217	1.75
Test requested for PM		230	1.64
Test request for coagulation		1547	12.47
Sample requested site	OPD	6207	50.05
	IPD	1193	9.62
	Emergency	730	5.89
	MCH	2,550	20.56
	Unknown addresses	1722	13.88
Sample collection shift	First (morning)	7109	57.32
	Second (afternoon)	5293	42.68
Type of sample collectors	Laboratory professionals	8255	66.56
	Non‐Laboratory healthy professionals	4147	33.44
Recording system	Manually	12,402	100
	laboratory information system	0	0

### 3.2 Overall Prevalence of Errors and Performance Levels By Sigma Metrics in Hematology Laboratory

The overall prevalence of hematology laboratory errors was 12.44%. Most errors occurred in the pre‐analytical phase with 43,148 errors (77.87%), followed by the post‐analytical phase with 10,954 (19.77%) and the analytical phase had 1305 (2.36%). The overall sigma value for the hematology laboratory was 2.7, with mean sigma values of 2.6, 3.2, and 2.8 for the pre‐analytical, analytical, and post‐analytical phases, respectively.

### 3.3 The Sigma Metrics Levels of the Pre‐Analytical Phase Related to Missed Information on Hematology Laboratory Requests

The sigma values for inappropriate and unauthorized test requests, missing MRN, and missed test orders were greater than three. The average sigma value for request form completeness was below three (Table 3).

**Table 3:** Sigma metrics performance levels of Hematology Laboratory request forms at the Dessie Comprehensive Specialized Hospital, Northeast Ethiopia, 2023.

Variables	No. of Error	Percentage	Total	Sigma value
Appropriate and authorized requests	721	5.81	12,402	3.1
MRN	268	2.16	12,402	3.5
Patient age	2671	21.54	12,402	2.3
Sex of patient	2277	18.36	12,402	2.5
Signature of the physician	4917	39.65	12,402	1.8
Clinical history of the patient	12402	100	12,402	< 3
Patients address	6239	50.31	12,402	1.5
Name of sender address/ward	1747	14.08	12,402	2.6
Date of order	4867	39.24	12,402	1.8
Test ordered	0	0	12,402	> 6.0
Time of sample collection	1854	14.95	12,402	2.6
Handwriting legible	984	7.93	12,402	3.0
Total	38,947	26.17	148,824	2.2

### 3.4 Medical Record Number (MRN)

#### 3.4.1 The Sigma Metrics Levels of Pre‐Analytical Phase Related to Specimen Quality, Collection, Preparation, Storage and Transportation in the Hematology Lab

In the assessment of specimen quality, 704 (5.68%) samples were deemed unsuitable for analysis. The frequencies of hemolyzed, incorrectly labeled, clotted, and insufficient samples were 173 (1.39%), 226 (1.82%), 258 (2.08%), and 363 (2.93%), respectively, each with sigma values greater than three. Additionally, the sigma metrics for lost test requests and lost samples were ≥ 4.2. Overall, the pre‐analytical performance level was below the three sigma value (Table 4).

**Table 4:** Sigma metrics levels of Hematology laboratory in pre‐analytical phases related to specimen quality, collection, preparation, storage and transportation, Dessie Comprehensive Specialized Hospital, Northeast, Ethiopia, 2023.

Variables	No. of error	Percentage	Total (N/%)	Sigma value
Hemolyzed samples	173	1.39	12,402	3.7
Clotted samples	258	2.08	12,402	3.6
Insufficient volume	363	2.93	12,402	3.4
Incorrect containers	0	0	12,402	> 6
Incorrectly labeled specimen	226	1.82	12,402	3.6
Delayed samples	116	0.94	12,402	3.9
Wrong sample transportation	52	0.42	12,402	4.2
Sample lost	45	0.36	12,402	4.2
Request lost	32	0.26	12,402	4.3
Unacceptable quality smear	24	10.43	230	2.8
Wrong sample storage	35	100	35	< 3
Blood mixed with an anticoagulant improperly	568	4.75	11,955	3.2
Improperly sealed capillary tube	21	9.68	217	2.9
Incorrect anticoagulant‐to‐blood ratio	1416	11.84	11,955	2.7
Patient identified improperly	234	2.32	10,076	3.5
Incorrect tourniquet application time	572	5.88	9721	3.1
Blood is unmixed before analysis	66	0.63	10,408	4.0
Total	4201	2.53	166,215	3.5
Sample unsuitable for analysis	704	5.68	12,402	3.1
Grand total pre‐analytical errors	43,148	13.70	315,039	2.6

### 3.5 Total Frequency (N)

#### 3.5.1 Sigma Metrics Levels of Analytical Phase in Hematology Laboratory

Among the 27,432 QIs assessed during the analytical phase, 1305 (4.79%) errors were identified. Nonlinear and questionable results with sigma values below three were reported without further testing or morphological examination. Internal quality control results passed consistently, with sigma values above 6. Overall, the analytical phase achieved a sigma value greater than 3 (Table 5).

**Table 5:** Sigma metrics levels of Hematology Laboratory in analytical phase at Dessie Comprehensive Specialized Hospital, Northeast Ethiopia, 2023.

Variables	No. of error	Percentage	Total	Sigma value
Daily IQC was not performed	42	72.73	66	1.2
IQC result failed	0	0	14	> 6
Background not checked	5	7.58	66	3.0
Preventive maintenance was not performed	17	25.76	81	2.1
Equipment malfunction observed	0	0	66	> 6
Reference range unavailable for parameters	0	0	18	> 6
Electric power inconsistency during analysis	521	4.81	10,823	3.2
Non‐linear results released without retesting	18	100	18	< 3
Reagents expired	2	3.03	66	3.4
Inappropriate reagent storage condition	0	0	66	> 6
Reagent stockout during analysis	218	2.06	10,606	3.6
Methods not updated upon the new reagent	0	0	12	> 6
Improperly filled ESR tube	44	8.37	526	2.9
The position of ESR tube is wrong	31	5.89	526	3.1
Delay in ESR results reading	38	7.22	526	3.0
ESR sample analyzed at the wrong temperature	0	0	526	> 6
Questionable results were not retested	79	26.16	302	2.2
Critical results were not checked by PM	98	68.53	143	1.1
HCT tube leaked	32	17.78	180	2.5
HCT tube broken	14	7.78	180	3.0
The speed of the centrifuge was adjusted improperly	6	3.33	180	3.4
The time of the centrifuge was adjusted improperly	7	3.89	180	3.3
HCT results were measured incorrectly	10	5.56	180	3.1
The smear was not properly air dried	5	2.34	213	3.5
Incorrect preparation of the working solution for PM	4	6.06	66	3.1
Smear stained at the incorrect time	79	37.09	213	1.9
Incorrectly washed smear	17	7.98	213	3.0
Cuvettes are not clean for coagulation	18	1.51	1189	3.7
Total	1305	4.79	27,245	3.2

#### 3.5.2 Sigma Metrics Level of Post‐Analytical Phase in Hematology Laboratory

Among the 103,261 QIs assessed in the post‐analytical phase, 10,954 (10.61%) errors were identified. Sigma values for failures to communicate critical results to physicians and releasing without verification were below 3. Overall, the mean sigma value for the post‐analytical phase was less than three (Table 6).

**Table 6:** Sigma metrics performance level of Hematology Laboratory in post analytical phase at Dessie Comprehensive Specialized Hospital, Northeast Ethiopia, 2023.

Variables	No. of error	Percentage	Total	Sigma value
Critical values were not communicated to the physician immediately	106	71.62	148	1.0
Results released without result verification	9867	85.90	11,487	0.5
Test results unrecorded	152	1.32	11,487	3.8
Results released without TAT	596	5.19	11,487	3.2
Result reported without standard unit	13	0.11	11,352	4.6
Samples were not retained/stored as per the policy	89	0.77	11,487	4.0
Laboratory results lost	49	0.43	11,487	4.2
Results reported with incorrect standard unit	0	0	11,352	> 6
Results reported without reference range	65	0.57	11,487	4.1
Result reported by unauthorized personnel	17	0.15	11,487	4.5
Total	10954	10.60807	103,261	2.8

### 3.6 Turnaround Time (TAT), Defect Per Million Opportunities (DPMO)

#### 3.6.1 Location of Sample Collection with Pre and Post‐Analytical Errors

Out of the 12,402 hematology test requests evaluated, 755 (6.09%) lacked patient age information. Among 704 rejected specimens, 71 (0.6%) hemolyzed samples requested from the emergency section. Of the 596 test results released beyond the expected TAT, 273 (2.38) were reported to the OPD (Table 7).

**Table 7:** Association of patient location and shift with pre‐analytical and post‐analytical errors at Dessie Comprehensive Specialized Hospital, 2023.

Variables	Location of patients (Clinic/Ward)				
	OPD	IPD	Emergency	MCH	Unknown
Patient age missed	753 (6.07)	232 (1.87)	471 (3.80)	460 (3.71)	755 (6.09)
Gender of patient missed	873 (7.04)	234 (1.89)	277 (2.23)	4 (0.03)	889 (7.71)
Signature of the physicians missed	1645 (13.26)	706 (5.69)	478 (3.85)	994 (8.01)	1094 (8.82)
Date of ordered missed	1690 (13.63)	550 (4.43)	481 (3.88)	902 (7.27)	1334 (10.76)
Hemolyzed specimen	38 (0.31)	71 (0.57)	32 (0.26)	12 (0.097)	19 (0.15)
Mislabeled specimen	34 (0.44)	89 (0.12)	61 (0.53)	24 (0.20)	18 (0.29)
Specimen rejection	293 (2.36)	107 (0.86)	45 (0.36)	133 (1.07)	126 (1.02)
Unrecorded results	42 (0.36)	28 (0.24)	64 (0.56)	6 (0.05)	12 (0.10)
Results released out of TAT	273 (2.38)	24 (0.21)	20 (0.17)	177 (1.54)	102 (0.89)

### 3.7 Factors Associated with Prolonged TAT

Factors associated with prolonged TAT were analyzed using a logistic regression model, with model goodness‐of‐fit was assessed by the Hosmer–Lemshow test (*p* = 0.056). In the bivariate logistic regression analysis, the first work shift (8.00 to 12:30 AM), patient location (IPD, OPD and unknown), and lack of adherence to SOP were significantly associated with the prolonged TAT. After adjusting for potential confounders, multivariate analysis identified that first shift (AOR: 1.92; 95% CI: 1.60–2.30; *p* < 0.001), IPD (AOR: 1.89; 95% CI: 1.62–2.32; *p* < 0.001), OPD (AOR: 1.98; 95% CI: 1.23–2.64; *p* < 0.001), unknown address (AOR: 2.23; 95% CI: 1.37–3.64; *p* < 0.001), and lack of adherence to SOPs (AOR: 2.88; 95% CI: 2.34–3.54; *p* < 0.001) as significant predictors of delayed TAT (Table 8).

**Table 8:** Logistic regression analysis of prolonged TAT and explanatory variables in clinical Hematology Laboratory at DCSH, Northeast Ethiopia, 2023.

Variable	Category	Prolonged TAT		COR (95% CI)	AOR (95% CI)	*P*‐value
		Yes	No			
Work shift	First	421	6061	2.14 (1.67–3.54)	1.92 (1.60–2.30)	< 0.001*
	Second	175	4830	1^a^	1^a^	1^a^
Ward	IPD	26	1044	2.56 (2.21–2.95)	1.89 (1.62–2.32)	< 0.001*
	OPD	272	5562	2.14 (1.39–3.257)	1.98 (1.23–2.64)	< 0.001*
	MCH	176	2141	1.45 (0.79–2.67)	1.35 (0.74–2.46)	0.23
	Unknown	102	1499	3.59 (2.33–5.54)	2.23 (1.37–3.64)	< 0.001*
	Emergency	20	645	1^a^	1^a^	1^a^
Lack of adherence to SOP	Yes	483	6509	3.35 (2.70–4.15)	2.88 (2.34–3.54)	< 0.001*
	No	113	4382	1^a^	1^a^	1^a^

* Stastical significance.

Reference Categories [1a], Crude Odds Ratio (COR), Confidence Interval (CI), Adjusted Odds ratio (AOR), Laboratory Information System (LIS), Inpatient Department (IPD), Outpatient Department (OPD) and Standard Operating Procedure (SOP).

### 3.8 Factors Associated with Sample Rejection

Factors contributing to specimen rejection were analyzed using a logistic regression model, with the model's goodness‐of‐fit assessed through the Hosmer–Lemeshow test (*p* = 0.631). In the bivariate logistic regression analysis, patient location (IPD and Unknown) and non‐adherence to SOPs were significantly associated with specimen rejection. The multivariate logistic regression analysis confirmed an independent association between IPD (AOR: 1.99; 95% CI: 1.58–2.50; *p* < 0.001), unknown address (AOR: 1.59; 95% CI: 1.28–1.98; *p* < 0.001), and non‐adherence to SOPs (AOR: 1.60; 95% CI: 1.38–1.86; *p* < 0.001) with specimen rejection (Table 9).

**Table 9:** Logistic regression analysis of sample rejection and explanatory variables in the clinical Hematology Laboratory at DCSH, Northeast Ethiopia, 2023.

Variable	Category	Rejection		COR (95% CI)	AOR (95% CI)	*P*‐value
		Yes	No			
Work shift	First	405	6704	1.01 (0.87–1.18)	0.95 (0.79–1.13)	0.58
	Second	299	4994	_1_a	_1_a	_1_a
Ward	IPD	107	1086	2.10 (1.66–2.66)	1.99 (1.58–2.50)	< 0.001*
	MCH	133	2417	1.10 (0.89–1.37)	1.11 (0.90–1.37)	0.33
	Emergency	45	685	1.93 (1.37–2.73)	1.33 (0.96–1.83)	0.09*
	Unknown	126	1596	2.25 (1.76–2.88)	1.59 (1.28–1.98)	< 0.001*
	OPD	293	5914	_1_a	_1_a	_1_a
Lack of adherence to SOP	Yes	302	4541	2.00 (1.69–2.37)	1.60 (1.38–1.86)	< 0.001*
	No	352	7207	_1_a	_1_a	_1_a

* Stastical significance.

Reference categories [1a], Crude odds ratio (COR), Confidence interval (CI) Adjusted Odds ratio (AOR), Inpatient Department (IPD), Outpatient Department (OPD) and Standard Operating Procedure (SOP).

## 4 Discussion

This study aimed to evaluate the performance of hematology laboratory in identifying and managing errors throughout the TTP. The findings revealed that the overall performance of the laboratory was suboptimal, with a sigma value of 2.7. This result is comparable to a study conducted in Gondar, Ethiopia, which reported a sigma value of 2.2 [42]. However, the performance of our laboratory was lower than that reported in studies from Tehran, Iran [1], Guangdong, China [43], India [44], Mysore, India [45] and Turkey [3], all of which reported performance levels exceeding three sigma. This discrepancy may be attributed to variations in QIs, test ordering systems, laboratory infrastructure, and the expertise and experience of healthcare professionals. Notably, the laboratories in these studies included fewer comprehensive QIs, had more advanced laboratories, and utilized an electronic system for ordering all tests, unlike the current study.

The poor performance of the Dessie hematology laboratory (sigma value < 3) may be attributed to several operational and systemic factors. Non‐adherence to standard operating procedures, limited training opportunities for laboratory personnel, the frequent collection of blood specimens by non‐laboratory professionals, and a high daily workload likely contribute to increased human errors. Moreover, the continued reliance on manual record‐keeping and did not use laboratory information systems increase the risk of mislabeling, sample loss, and delayed result reporting. The absence of regular internal quality control for hematology tests and the laboratory's limited participation in external quality assessment programs further hinder continuous quality improvement. To enhance laboratory performance, several practical and sustainable interventions can be implemented. Regular in service training should be provided to strengthen staff competency and ensure strict adherence to standard operating procedures. Assigning specimen collection exclusively to trained laboratory professionals would reduce pre‐analytical errors. Introducing a computerized laboratory information management system can minimize transcription errors and improve traceability of samples and results. Furthermore, establishing a robust internal quality control system for all hematological tests, coupled with consistent participation in national or international external quality assessment schemes, would enhance analytical reliability. Furthermore, adopting Lean and Six Sigma quality management approaches could help reduce variation and optimize workflow efficiency.

In this study, the overall prevalence of clinical hematology laboratory errors in the TTP was 12.44%, with pre‐analytical, analytical, and post‐analytical error rates of 9.68%, 0.29%, and 2.46%, respectively. The finding is comparable to studies conducted in Dessie, Ethiopia [11] and Aurangabad, India [46], which reported overall error rates of 15.3% and14.9%, respectively. However, it is higher than studies conducted in Anokye, Ghana [47], Assah, Saudi Arabia [48], Teerthanker Mahaveer, India [49], Imam, Iran [50] and Lahore, Pakistan [51], which reported total error rates of 4.7%, 4.35%, 0.17%, 6.3%, and 1.2%, respectively. Conversely, the overall error frequency in our study was lower than hat reported in Gondar, Ethiopia [42], Gondar, Ethiopia [52], Addis Ababa [24] and Wolega, Ethiopia [53], which reported defect rates of 26%, 36.8, 28.5% and 58.2%, respectively. The discrepancy among these findings could be attributed to differences in the QIs included and the operational definitions used to assess errors. Additionally, variations in the sample size, technical capacity and the inclusion of samples from different laboratory units rather than focusing solely on hematological tests, may have contributed to the observed differences.

In the pre‐analytical phase, the mean sigma value was below the acceptable limit ( < 3 sigma). Possible root causes for this lower performance include inadequate patient preparation, improper sample collection techniques, insufficient mixing of anticoagulated blood, and improper transportation or storage of specimens. Additionally, factors such as limited experience, negligence, lack of attention, high workload, and non‐compliance with standard operating procedures (SOPs) may have contributed to inconsistencies. In contrast, the performance of our laboratory in the analytical and post‐analytical phases was marginal (3.2) and poor (2.8), respectively. The lower performance in the analytical phase may be attributed to a shortage of trained personnel, inadequate reagent supply, insufficient preventive maintenance, a small sample size, and a lack of verification and awareness regarding critical value checks. Poor performance in the post‐analytical phase could be due to failure to communicate critical test results to physicians, unverified test results, and delayed release of results beyond the established turnaround time (TAT). Additional contributing factors may include increased workload, weak implementation of laboratory policies, an inefficient reporting system, inadequate infrastructure, and electrical fluctuations. In this study, the sigma value for incomplete filling of test request forms was below the acceptable limit ( < 3). This finding is similar to a study done Gondar, Ethiopia, which also reported a sigma value below 3 [42]. However, our performance was lower than that reported in studies conducted in India [54] and Romanian [43], which reported optimal performance ( > 3) which observed optimal performance ( > 3). This variation could be attributed to differences in the experience and consistency of physicians attending to patients at the study site, as most of the physicians were medical students.

According to the current study, the laboratory achieved undesirable performance in specimen quality, with a sigma value of 2.6, indicating that performance in this area was lower than that reported in a study conducted in eastern India ( > 4 sigma) [55]. The lower performance in our laboratory may be attributed to non‐adherence to standard operating procedures (SOPs), reliance on manual recording systems, work overload, and sample collection by untrained personnel.

On the other hand, the laboratory achieved marginal performance (3.4–3.7 sigma) for hemolysis, wrong labeling, clotting, and incorrect sample volume. Although these values are within the acceptable limit, they do not represent world‐class performance. Studies conducted in India [54], China [43], Romania [43] and the United States [56] reported better performance (very good to world‐class) for these parameters. The lower performance observed in our study could be due to improper tourniquet application during sample collection, insufficient mixing of blood with anticoagulant, negligence, and suboptimal sample transportation conditions.

In this study, the performance of verifying nonlinear results through retesting and examining questionable results by morphology was undesirable ( < 3 sigma). This indicates that our laboratory's performance is lower than studies conducted in Guangdong, China [43], Mysore, India [45] and Turkey [3], where performance levels were reported as desirable. The poor performance in our laboratory may be due to a lack of verification and awareness regarding the importance of rechecking critical values.

The current study also indicated poor performance ( ≤ 3 sigma) in communicating critical test results to physicians, verifying test results, and releasing results within the established turnaround time (TAT). Our laboratory's performance in these areas is lower than that reported in China [43], India [45] and Turkey [3]. Contributing factors may include increased workload, weak laboratory policies, inefficient reporting systems, inadequate infrastructure, and electrical fluctuations.

Multivariate logistic regression analysis revealed that prolonged TAT was significantly associated with sample collection from unknown wards (AOR = 2.23, 95% CI: 1.37–3.64), IPD (AOR = 1.89, 95% CI: 1.62–2.32), OPD (AOR = 1.98, 95% CI: 1.23–2.64), the first work shift (AOR = 1.92, 95% CI: 1.60–2.30), and lack of adherence to SOPs (AOR = 2.88, 95% CI: 2.34–3.54). These findings are consistent with studies conducted in Gondar, Ethiopia (2023) [42] and the Armed Force hospital, Ethiopia [57]. Prolonged TAT during the first (morning) shift may be due to work overload and fatigue, while delays associated with samples from unknown wards, IPD, and OPD may relate to the physical distance between sample collection sites and the analysis room. In the study area, samples were collected from wards located far from the laboratory.

Furthermore, the study showed that patient address (IPD and unknown) and non‐adherence to SOPs were significantly associated with unsuitable samples. Lack of adherence to SOPs nearly doubled the likelihood of sample rejection (AOR = 1.67, 95% CI: 1.38–1.86) compared to adherence, consistent with findings from Gondar, Ethiopia (2023) [42] and Kenya [58]. Poor SOP adherence in our setting may be due to weak standardization among laboratory personnel, sample collection by non‐laboratory staff, work overload, and inadequate supervision. Similarly, IPD samples had nearly twice the rejection rate (AOR = 1.99, 95% CI: 1.58–2.50) compared to OPD samples, a finding supported by studies in Gondar, Ethiopia (2023) [42], Hawassa, Ethiopia [59], Nepal [60] and India [46]. Samples from unknown addresses also had nearly twice the rejection rate (AOR = 1.59, 95% CI: 1.28–1.98) compared to OPD. This may be related to sample collection by untrained non‐laboratory personnel.

### 4.1 Strengths and Limitations of the Study

The strength of this study lies in its comprehensive assessment of the TTP performance of the Clinical Hematology Laboratory using a relatively detailed checklist. Additionally, sigma metrics were employed as the performance assessment tool, providing an objective measure of laboratory quality across all TTP phases. However, the study has some limitations. Hemolysis was assessed visually, which may have introduced interpersonal bias. The study was conducted over a relatively short period, and samples collected outside normal working hours were not included. Furthermore, not all potential root causes of low performance were evaluated. Finally, the performance of the automated hematology analyzer could not be assessed using sigma metrics due to the absence of regular quality control.

## 5 Conclusion and Recommendation

### 5.1 Conclusion

This study concluded that clinical hematology laboratory errors occurred at a high frequency, with the majority detected during the pre‐analytical phase, followed by the post‐analytical phase. Analytical errors were the least frequent. Among pre‐analytical errors, incomplete request forms and unsuitable samples were the most common. Lack of adherence to SOPs and patient addresses from IPD and unknown wards were significantly associated with poor sample quality. Similarly, the first work shift, IPD, and unknown addresses were significantly associated with prolonged turnaround time (TAT). Overall, the Hematology Laboratory demonstrated poor sigma metric values (< 3 sigma), indicating that the quality of services provided was not assured.

### 5.2 Recommendations

We recommend conducting further studies with an extended study period and involving a larger number of laboratory and healthcare professionals. For Dessie Comprehensive Specialized Hospital, we suggest providing targeted training for healthcare professionals on proper sample handling and implementing immediate corrective actions in the TTP, prioritizing improvements in the pre‐analytical phase. The hospital should consider sigma metrics as a tool for ongoing quality monitoring and improvement. Laboratory personnel are advised to strictly follow SOPs across all phases of the testing process. Overall, the laboratory should implement regular and comprehensive internal and external audits to identify deficiencies and take evidence‐based corrective measures.

## Author Contributions

**Zewudu Mulatie:** conceptulization, project administration, writing – orginal draft and writing – review and editing the manuscript. **Endris Ebrahim:** formal analysis and validation. **Mihreteab Alebachew:** methodology, softwarre and investigation. **Alemu Gedefie:** formal analysis, supervision. **Bruktawit Eshetu:** formal analysis, validation and software. **Mihret Tilahun:** software, supervision, data curation. **Habtu Debash:** investigation, resources, software. **Yeshimebet Kassa:** visualization, investigation and resource. **Ermiyas Alemayehu:** formal analysis, validation and data curation. **Tesfaye Gessese:** Investigation, data curation and methodology. **Dereje Mengesha Berta:** formal analysis, project administration, validation and writing – review and editing the manuscript. All authors reviewed and approved the manuscript.

## Funding

The authors received no specific funding for this work.

## Ethics Statement

Ethical approval was obtained from the Ethical Review Committee of the College of Medicine and Health Sciences, according to the declaration of Helsinki. The corresponding author, Zewudu Mulatie, had full access to all of the data in this study and took complete responsibility for the integrity of the data and the accuracy of the data analysis.

## Conflicts of Interest

The authors declare that there are no conflicts of interest regarding the publication of this manuscript.

## Consent for Publication

The authors have nothing to report.

## Transparency Statement

The lead author, Zewudu Mulatie, affirms that this manuscript is an honest, accurate, and transparent account of the study being reported; that no important aspects of the study have been omitted; and that any discrepancies from the study as planned (and, if relevant, registered) have been explained.

## Supporting information

Supporting 2 STROBE‐checklist.

Supporting file 1: Raw data summary.

## Data Availability

The data supporting these findings are contained within the manuscript. If additional data are needed, it can be obtained from the corresponding authors upon request.
