# Supplementary material for: Assessment of Clinical Hematology Laboratory Performance Using Sigma Metrics and Associated Factors in Total Testing Process at the Dessie Comprehensive Specialized Hospital, Northeast, Ethiopia: A Cross‐Sectional Study
Source: Health Sci Rep. 2026 Mar 10;9(3):e72063. doi: 10.1002/hsr2.72063 (PMC12975641; doi:10.1002/hsr2.72063)
Supplement: Supplementary file 2 — Supporting file 1: Raw data summary. [file HSR2-9-e72063-s001.docx]

Supplementary file 1: raw data summaries that show sigma value

Frequency of errors and sigma metrics performance levels of Hematology Laboratory request forms at the Dessie Comprehensive Specialized Hospital, Northeast Ethiopia, 2023

| Variables | No. of Error/ defects | Percentage | Total | DPMO | Sigma value |
| --- | --- | --- | --- | --- | --- |
| Appropriate and authorized requests | 721 | 5.81 | 12,402 | 58136 | 3.1 |
| MRN | 268 | 2.16 | 12,402 | 23061 | 3.5 |
| Patient age | 2671 | 21.54 | 12,402 | 215368 | 2.3 |
| Sex of patient | 2277 | 18.36 | 12,402 | 183599 | 2.5 |
| Signature of the physician | 4917 | 39.65 | 12,402 | 396468 | 1.8 |
| Clinical history of the patient | 12402 | 100 | 12,402 | 1000000 | <3 |
| Patients address | 6239 | 50.31 | 12,402 | 503064 | 1.5 |
| Name of sender address/ward | 1747 | 14.08 | 12,402 | 140864 | 2.6 |
| Date of ordered | 4867 | 39.24 | 12,402 | 392437 | 1.8 |
| Test ordered | 0 | 0 | 12,402 | 0 | >6.0 |
| Time of sample collection | 1854 | 14.95 | 12,402 | 149492 | 2.6 |
| Handwriting legible | 984 | 7.93 | 12,402 | 79342 | 3.0 |
| Total | 38,947 | 26.17 | 148,824 | 261698 | 2.2 |

Medical record number (MRN), Defect per million opportunities (DPMO)

Frequency errors and sigma metrics levels of Hematology laboratory in pre analytical phases related with specimen quality, collection, preparation, storage and transportation, Dessie Comprehensive Specialized Hospital, Northeast, Ethiopia, 2023

| Variables | No. of error | Percentage | Total (N/%) | DPMO | Sigma  value |
| --- | --- | --- | --- | --- | --- |
| Hemolyzed samples | 173 | 1.39 | 12,402 | 13949 | 3.7 |
| Clotted samples | 258 | 2.08 | 12,402 | 20803 | 3.6 |
| Insufficient volume | 363 | 2.93 | 12,402 | 29269 | 3.4 |
| Incorrect containers | 0 | 0 | 12,402 | 0 | >6 |
| Incorrectly labeled specimen | 226 | 1.82 | 12,402 | 18223 | 3.6 |
| Delayed samples | 116 | 0.94 | 12,402 | 9353 | 3.9 |
| Wrong sample transportation | 52 | 0.42 | 12,402 | 4193 | 4.2 |
| Sample lost | 45 | 0.36 | 12,402 | 3628 | 4.2 |
| Request lost | 32 | 0.26 | 12,402 | 2580 | 4.3 |
| Unacceptable quality smear | 24 | 10.43 | 230 | 104348 | 2.8 |
| Wrong sample storage | 35 | 100 | 35 | 1000000 | <3 |
| Blood mixed with anticoagulant improperly | 568 | 4.75 | 11,955 | 47512 | 3.2 |
| Improperly sealed capillary tube | 21 | 9.68 | 217 | 96774 | 2.9 |
| Incorrect anticoagulant to blood ratio | 1,416 | 11.84 | 11,955 | 118444 | 2.7 |
| Patient identified improperly | 234 | 2.32 | 10,076 | 23224 | 3.5 |
| Incorrect tourniquet application time | 572 | 5.88 | 9,721 | 58842 | 3.1 |
| Blood unmixed before analysis | 66 | 0.63 | 10,408 | 6341 | 4.0 |
| Total | 4,201 | 2.53 | 166,215 | 25274 | 3.5 |
| Sample unsuitable for analysis | 704 | 5.68 | 12,402 | 56765 | 3.1 |
| Grand total pre-analytical errors | 43,148 | 13.70 | 315,039 | 136961 | 2.6 |

Total frequency (N) Defect per million opportunities (DPMO)

Frequency of errors and the sigma metrics levels of Hematology Laboratory in analytical phase at Dessie Comprehensive Specialized Hospital, Northweast Ethiopia, 2023

| Variables | No. of error | Percentage | Total | DPMO | Sigma  Value |
| --- | --- | --- | --- | --- | --- |
| Daily IQC not performed | 42 | 72.73 | 66 | 636364 | 1.2 |
| IQC result failed | 0 | 0 | 14 | 0 | >6 |
| Background not checked | 5 | 7.58 | 66 | 75758 | 3.0 |
| Preventive maintenance not performed | 17 | 25.76 | 81 | 209877 | 2.1 |
| Equipment mal-functionality observed | 0 | 0 | 66 | 0 | >6 |
| Reference range unavailable for parameters | 0 | 0 | 18 | 0 | >6 |
| Electric power inconsistence during analysis | 521 | 4.81 | 10,823 | 48138 | 3.2 |
| Non-linear results released without retesting | 18 | 100 | 18 | 1000000 | <3 |
| Reagents expired | 2 | 3.03 | 66 | 30303 | 3.4 |
| Inappropriate reagent storage condition | 0 | 0 | 66 | 0 | >6 |
| Reagent stock out during analysis | 218 | 2.06 | 10,606 | 20554 | 3.6 |
| Methods not updated upon new reagent | 0 | 0 | 12 | 0 | >6 |
| Improperly filled ESR tube | 44 | 8.37 | 526 | 83650 | 2.9 |
| Position of ESR tube wrong | 31 | 5.89 | 526 | 58935 | 3.1 |
| Delay in ESR results reading | 38 | 7.22 | 526 | 72243 | 3.0 |
| ESR sample analyzed at wrong temperature | 0 | 0 | 526 | 0 | >6 |
| Questionable results were not retested | 79 | 26.16 | 302 | 261589 | 2.2 |
| Critical results were not checked by PM | 98 | 68.53 | 143 | 685315 | 1.1 |
| HCT tube leaked | 32 | 17.78 | 180 | 177778 | 2.5 |
| HCT tube broken | 14 | 7.78 | 180 | 77778 | 3.0 |
| Speed of centrifuge adjusted improperly | 6 | 3.33 | 180 | 33333 | 3.4 |
| Time of centrifuge adjusted improperly | 7 | 3.89 | 180 | 38889 | 3.3 |
| HCT results measured incorrectly | 10 | 5.56 | 180 | 55556 | 3.1 |
| Smear not properly air dried | 5 | 2.34 | 213 | 23474 | 3.5 |
| Incorrect preparation of working solution for PM | 4 | 6.06 | 66 | 60606 | 3.1 |
| Smear stained at incorrect time | 79 | 37.09 | 213 | 370892 | 1.9 |
| Incorrectly washed smear | 17 | 7.98 | 213 | 79812 | 3.0 |
| Cuvettes not clean for coagulation | 18 | 1.51 | 1,189 | 15139 | 3.7 |
| Total | 1305 | 4.79 | 27,245 | 47899 | 3.2 |

Frequency of errors and sigma metrics performance level of Hematology Laboratory in post analytical phase at Dessie Comprehensive Specialized Hospital, Northeast Ethiopia, 2023

| Variables | No. of error | Percentage | Total | DPMO | Sigma value |
| --- | --- | --- | --- | --- | --- |
| Critical values were not communicated to physician immediately | 106 | 71.62 | 148 | 716216 | 1.0 |
| Results released without result verification | 9,867 | 85.90 | 11,487 | 858971 | 0.5 |
| Test results unrecorded | 152 | 1.32 | 11,487 | 13232 | 3.8 |
| Results released without TAT | 596 | 5.19 | 11,487 | 51885 | 3.2 |
| Result reported without standard unit | 13 | 0.11 | 11,352 | 1145 | 4.6 |
| Samples were not retained/stored as the policy | 89 | 0.77 | 11,487 | 7748 | 4.0 |
| Laboratory results lost | 49 | 0.43 | 11,487 | 4266 | 4.2 |
| Results reported with incorrect standard unit | 0 | 0 | 11,352 | 0 | >6 |
| Results reported without reference range | 65 | 0.57 | 11,487 | 5659 | 4.1 |
| Result reported by unauthorized personnel | 17 | 0.15 | 11,487 | 1480 | 4.5 |
| Total | 10954 | 10.60807 | 103,261 | 106081 | 2.8 |

Turnaround time (TAT), Defect per million opportunities (DPMO)
